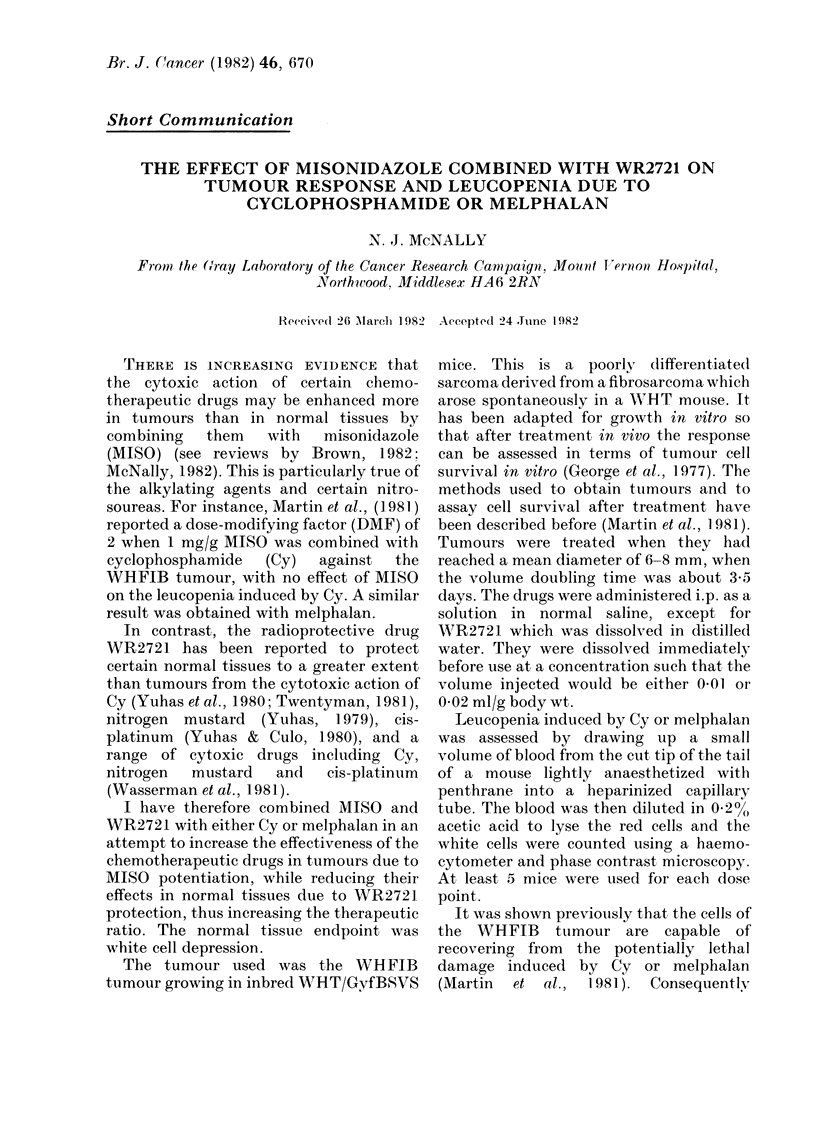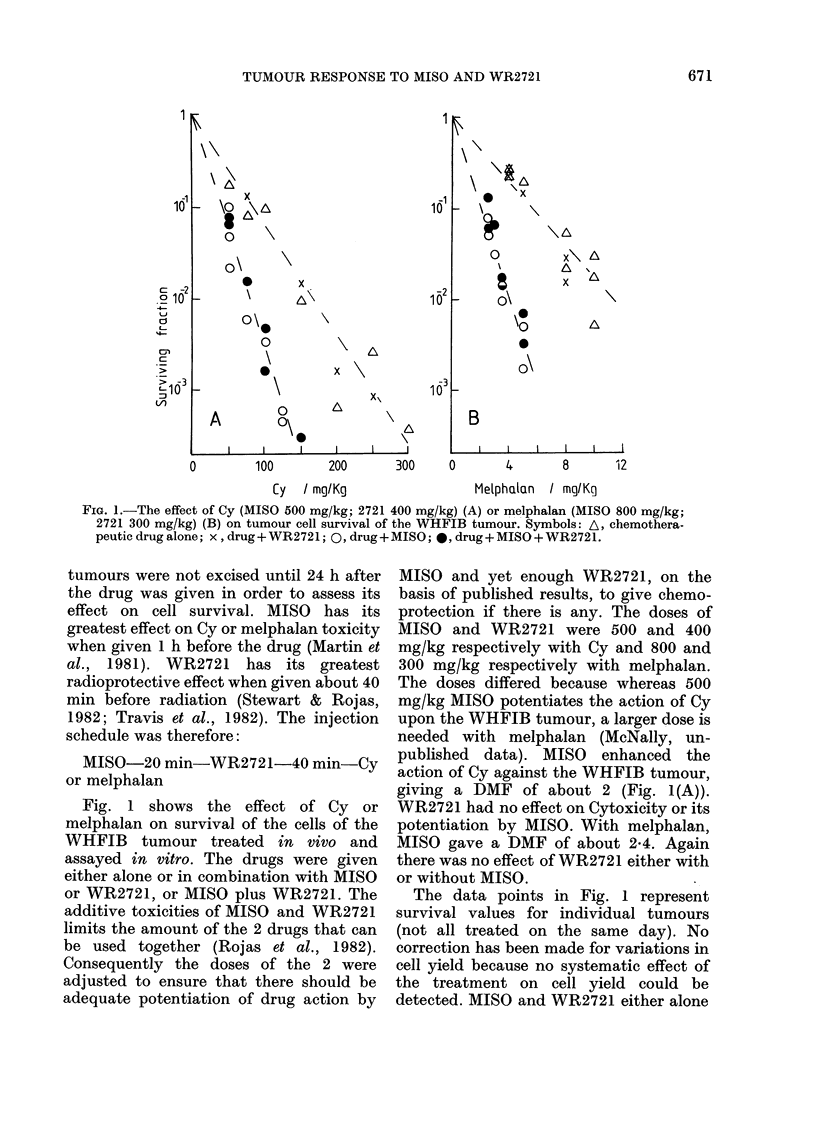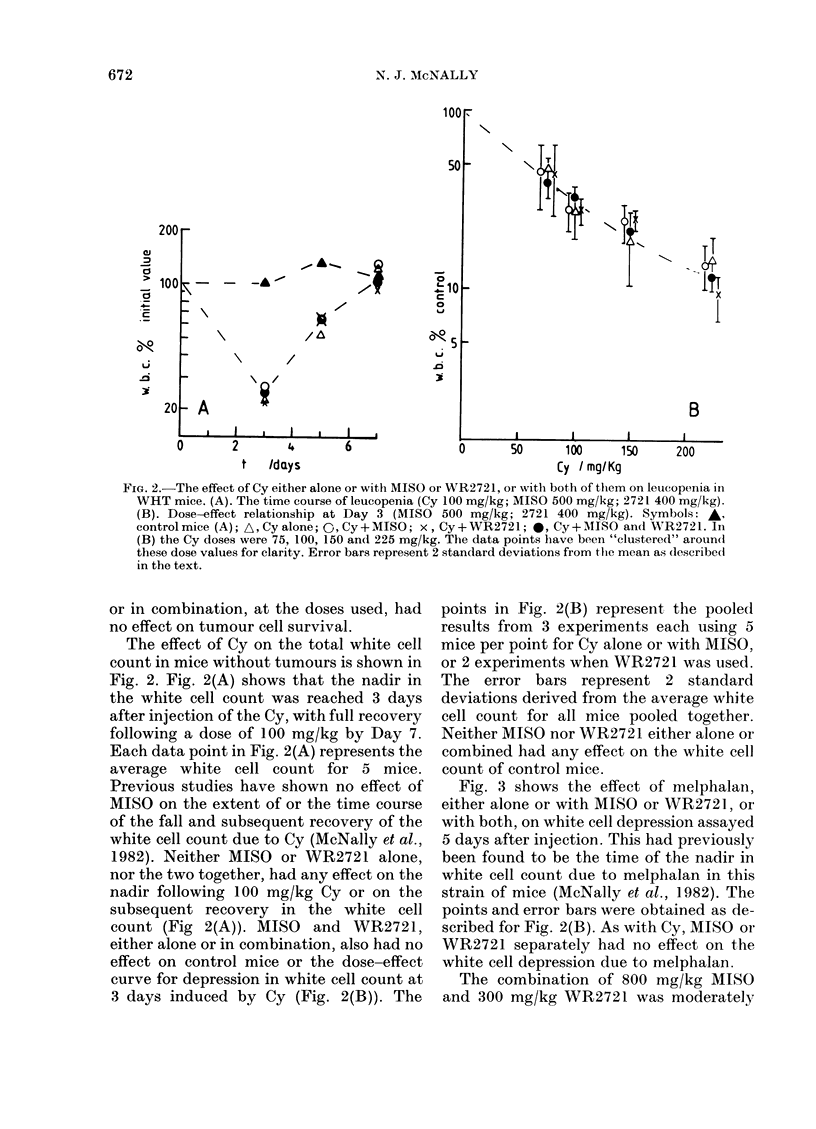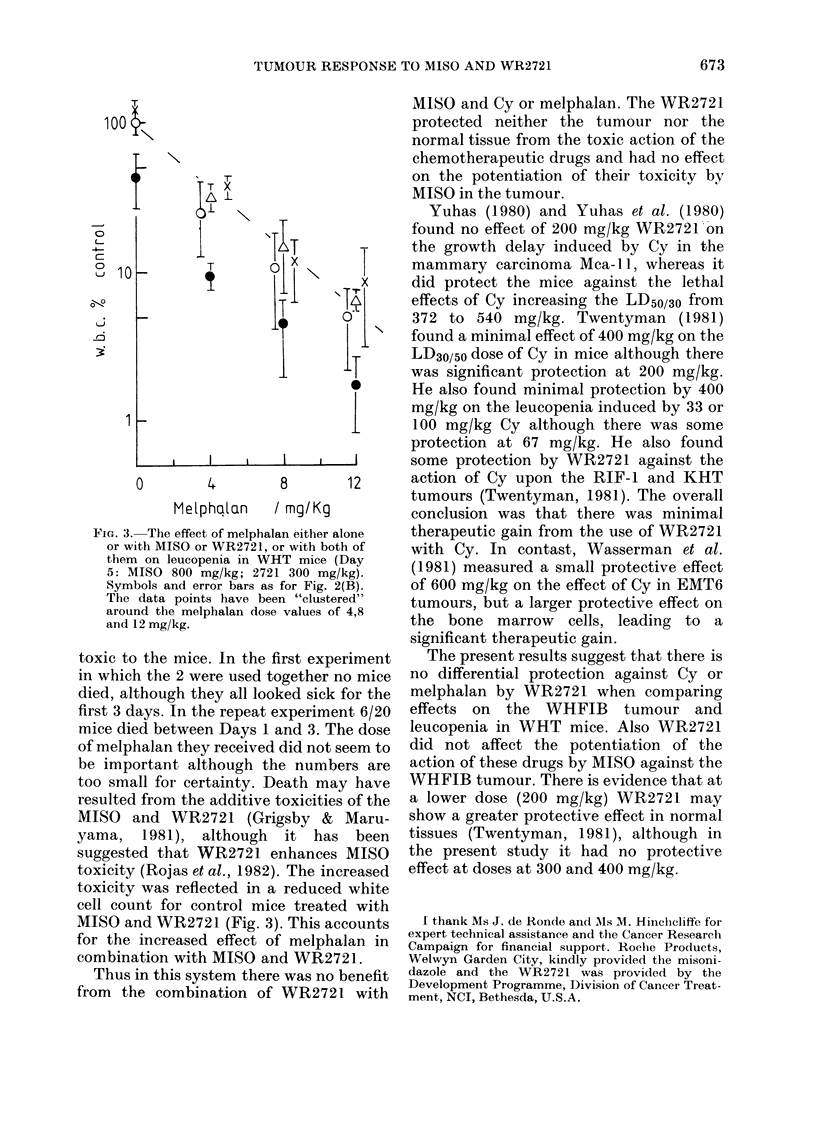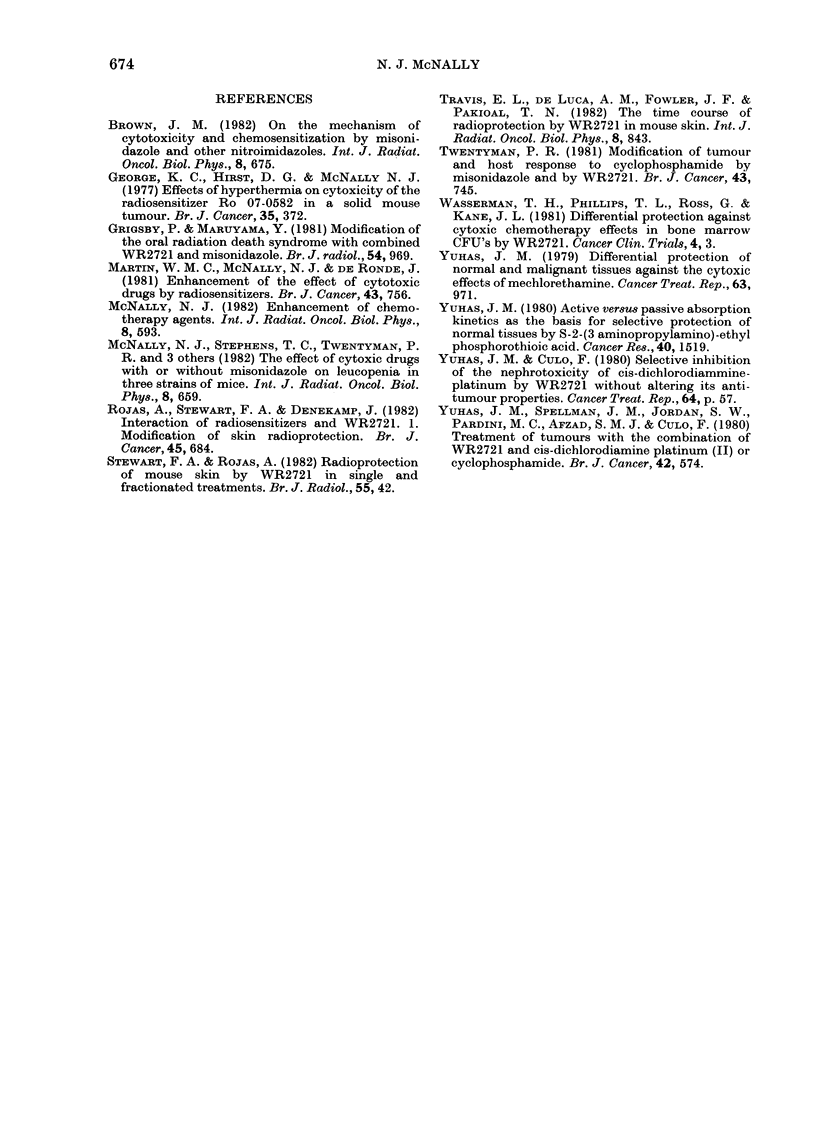# The effect of misonidazole combined with WR2721 on tumour response and leucopenia due to cyclophosphamide or melphalan.

**DOI:** 10.1038/bjc.1982.253

**Published:** 1982-10

**Authors:** N. J. McNally


*Br. J. Cancer* (1982) **46**, 670

***Short Communication***

### THE EFFECT OF MISONIDAZOLE COMBINED WITH WR2721 ON TUMOUR RESPONSE AND LEUCOPENIA DUE TO CYCLOPHOSPHAMIDE OR MELPHALAN

N. J. McNALLY

*From the Gray Laboratory of the Cancer Research Campaign, Mount Vernon Hospital, Northwood, Middlesex HA6 2RN*

Received 26 March 1982 Accepted 24 June 1982

THERE IS INCREASING EVIDENCE that the cytoxic action of certain chemotherapeutic drugs may be enhanced more in tumours than in normal tissues by combining them with misonidazole (MISO) (see reviews by Brown, 1982; McNally, 1982). This is particularly true of the alkylating agents and certain nitrosoureas. For instance, Martin *et al.*, (1981) reported a dose-modifying factor (DMF) of 2 when 1 mg/g MISO was combined with cyclophosphamide (Cy) against the WHFIB tumour, with no effect of MISO on the leucopenia induced by Cy. A similar result was obtained with melphalan.

In contrast, the radioprotective drug WR2721 has been reported to protect certain normal tissues to a greater extent than tumours from the cytotoxic action of Cy (Yuhas *et al.*, 1980; Twentyman, 1981), nitrogen mustard (Yuhas, 1979), cis-platinum (Yuhas & Culo, 1980), and a range of cytoxic drugs including Cy, nitrogen mustard and cis-platinum (Wasserman *et al.*, 1981).

I have therefore combined MISO and WR2721 with either Cy or melphalan in an attempt to increase the effectiveness of the chemotherapeutic drugs in tumours due to MISO potentiation, while reducing their effects in normal tissues due to WR2721 protection, thus increasing the therapeutic ratio. The normal tissue endpoint was white cell depression.

The tumour used was the WHFIB tumour growing in inbred WHT/GyfBSVS mice. This is a poorly differentiated sarcoma derived from a fibrosarcoma which arose spontaneously in a WHT mouse. It has been adapted for growth *in vitro* so that after treatment *in vivo* the response can be assessed in terms of tumour cell survival *in vitro* (George *et al.*, 1977). The methods used to obtain tumours and to assay cell survival after treatment have been described before (Martin *et al.*, 1981). Tumours were treated when they had reached a mean diameter of 6–8 mm, when the volume doubling time was about 3·5 days. The drugs were administered i.p. as a solution in normal saline, except for WR2721 which was dissolved in distilled water. They were dissolved immediately before use at a concentration such that the volume injected would be either 0·01 or 0·02 ml/g body wt.

Leucopenia induced by Cy or melphalan was assessed by drawing up a small volume of blood from the cut tip of the tail of a mouse lightly anaesthetized with penthrane into a heparinized capillary tube. The blood was then diluted in 0·2% acetic acid to lyse the red cells and the white cells were counted using a haemocytometer and phase contrast microscopy. At least 5 mice were used for each dose point.

It was shown previously that the cells of the WHFIB tumour are capable of recovering from the potentially lethal damage induced by Cy or melphalan (Martin *et al.*, 1981). Consequently

FIG. 1.—The effect of Cy (MISO 500 mg/kg; 2721 400 mg/kg) (A) or melphalan (MISO 800 mg/kg; 2721 300 mg/kg) (B) on tumour cell survival of the WHFIB tumour. Symbols: △, chemotherapeutic drug alone; ×, drug+WR2721; ○, drug+MISO; ●, drug+MISO+WR2721.

tumours were not excised until 24 h after the drug was given in order to assess its effect on cell survival. MISO has its greatest effect on Cy or melphalan toxicity when given 1 h before the drug (Martin *et al.*, 1981). WR2721 has its greatest radioprotective effect when given about 40 min before radiation (Stewart & Rojas, 1982; Travis *et al.*, 1982). The injection schedule was therefore:

MISO—20 min—WR2721—40 min—Cy or melphalan

Fig. 1 shows the effect of Cy or melphalan on survival of the cells of the WHFIB tumour treated *in vivo* and assayed *in vitro*. The drugs were given either alone or in combination with MISO or WR2721, or MISO plus WR2721. The additive toxicities of MISO and WR2721 limits the amount of the 2 drugs that can be used together (Rojas *et al.*, 1982). Consequently the doses of the 2 were adjusted to ensure that there should be adequate potentiation of drug action by MISO and yet enough WR2721, on the basis of published results, to give chemoprotection if there is any. The doses of MISO and WR2721 were 500 and 400 mg/kg respectively with Cy and 800 and 300 mg/kg respectively with melphalan. The doses differed because whereas 500 mg/kg MISO potentiates the action of Cy upon the WHFIB tumour, a larger dose is needed with melphalan (McNally, unpublished data). MISO enhanced the action of Cy against the WHFIB tumour, giving a DMF of about 2 (Fig. 1(A)). WR2721 had no effect on Cytoxicity or its potentiation by MISO. With melphalan, MISO gave a DMF of about 2·4. Again there was no effect of WR2721 either with or without MISO.

The data points in Fig. 1 represent survival values for individual tumours (not all treated on the same day). No correction has been made for variations in cell yield because no systematic effect of the treatment on cell yield could be detected. MISO and WR2721 either alone

FIG. 2.—The effect of Cy either alone or with MISO or WR2721, or with both of them on leucopenia in WHT mice. (A). The time course of leucopenia (Cy 100 mg/kg; MISO 500 mg/kg; 2721 400 mg/kg). (B). Dose–effect relationship at Day 3 (MISO 500 mg/kg; 2721 400 mg/kg). Symbols: ▲, control mice (A); △, Cy alone; ○, Cy+MISO; ×, Cy+WR2721; ●, Cy+MISO and WR2721. In (B) the Cy doses were 75, 100, 150 and 225 mg/kg. The data points have been "clustered" around these dose values for clarity. Error bars represent 2 standard deviations from the mean as described in the text.

or in combination, at the doses used, had no effect on tumour cell survival.

The effect of Cy on the total white cell count in mice without tumours is shown in Fig. 2. Fig. 2(A) shows that the nadir in the white cell count was reached 3 days after injection of the Cy, with full recovery following a dose of 100 mg/kg by Day 7. Each data point in Fig. 2(A) represents the average white cell count for 5 mice. Previous studies have shown no effect of MISO on the extent of or the time course of the fall and subsequent recovery of the white cell count due to Cy (McNally *et al.*, 1982). Neither MISO or WR2721 alone, nor the two together, had any effect on the nadir following 100 mg/kg Cy or on the subsequent recovery in the white cell count (Fig 2(A)). MISO and WR2721, either alone or in combination, also had no effect on control mice or the dose–effect curve for depression in white cell count at 3 days induced by Cy (Fig. 2(B)). The points in Fig. 2(B) represent the pooled results from 3 experiments each using 5 mice per point for Cy alone or with MISO, or 2 experiments when WR2721 was used. The error bars represent 2 standard deviations derived from the average white cell count for all mice pooled together. Neither MISO nor WR2721 either alone or combined had any effect on the white cell count of control mice.

Fig. 3 shows the effect of melphalan, either alone or with MISO or WR2721, or with both, on white cell depression assayed 5 days after injection. This had previously been found to be the time of the nadir in white cell count due to melphalan in this strain of mice (McNally *et al.*, 1982). The points and error bars were obtained as described for Fig. 2(B). As with Cy, MISO or WR2721 separately had no effect on the white cell depression due to melphalan.

The combination of 800 mg/kg MISO and 300 mg/kg WR2721 was moderately

FIG. 3.—The effect of melphalan either alone or with MISO or WR2721, or with both of them on leucopenia in WHT mice (Day 5: MISO 800 mg/kg; 2721 300 mg/kg). Symbols and error bars as for Fig. 2(B). The data points have been "clustered" around the melphalan dose values of 4,8 and 12 mg/kg.

toxic to the mice. In the first experiment in which the 2 were used together no mice died, although they all looked sick for the first 3 days. In the repeat experiment 6/20 mice died between Days 1 and 3. The dose of melphalan they received did not seem to be important although the numbers are too small for certainty. Death may have resulted from the additive toxicities of the MISO and WR2721 (Grigsby & Maruyama, 1981), although it has been suggested that WR2721 enhances MISO toxicity (Rojas *et al.*, 1982). The increased toxicity was reflected in a reduced white cell count for control mice treated with MISO and WR2721 (Fig. 3). This accounts for the increased effect of melphalan in combination with MISO and WR2721.

Thus in this system there was no benefit from the combination of WR2721 with MISO and Cy or melphalan. The WR2721 protected neither the tumour nor the normal tissue from the toxic action of the chemotherapeutic drugs and had no effect on the potentiation of their toxicity by MISO in the tumour.

Yuhas (1980) and Yuhas *et al.* (1980) found no effect of 200 mg/kg WR2721 on the growth delay induced by Cy in the mammary carcinoma Mca-11, whereas it did protect the mice against the lethal effects of Cy increasing the $LD_{50/30}$ from 372 to 540 mg/kg. Twentyman (1981) found a minimal effect of 400 mg/kg on the $LD_{30/50}$ dose of Cy in mice although there was significant protection at 200 mg/kg. He also found minimal protection by 400 mg/kg on the leucopenia induced by 33 or 100 mg/kg Cy although there was some protection at 67 mg/kg. He also found some protection by WR2721 against the action of Cy upon the RIF-1 and KHT tumours (Twentyman, 1981). The overall conclusion was that there was minimal therapeutic gain from the use of WR2721 with Cy. In contast, Wasserman *et al.* (1981) measured a small protective effect of 600 mg/kg on the effect of Cy in EMT6 tumours, but a larger protective effect on the bone marrow cells, leading to a significant therapeutic gain.

The present results suggest that there is no differential protection against Cy or melphalan by WR2721 when comparing effects on the WHFIB tumour and leucopenia in WHT mice. Also WR2721 did not affect the potentiation of the action of these drugs by MISO against the WHFIB tumour. There is evidence that at a lower dose (200 mg/kg) WR2721 may show a greater protective effect in normal tissues (Twentyman, 1981), although in the present study it had no protective effect at doses at 300 and 400 mg/kg.

I thank Ms J. de Ronde and Ms M. Hinchcliffe for expert technical assistance and the Cancer Research Campaign for financial support. Roche Products, Welwyn Garden City, kindly provided the misonidazole and the WR2721 was provided by the Development Programme, Division of Cancer Treatment, NCI, Bethesda, U.S.A.

### REFERENCES

BROWN, J. M. (1982) On the mechanism of cytotoxicity and chemosensitization by misonidazole and other nitroimidazoles. *Int. J. Radiat. Oncol. Biol. Phys.*, **8**, 675.

GEORGE, K. C., HIRST, D. G. & MCNALLY N. J. (1977) Effects of hyperthermia on cytoxicity of the radiosensitizer Ro 07-0582 in a solid mouse tumour. *Br. J. Cancer*, **35**, 372.

GRIGSBY, P. & MARUYAMA, Y. (1981) Modification of the oral radiation death syndrome with combined WR2721 and misonidazole. *Br. J. radiol.*, **54**, 969.

MARTIN, W. M. C., MCNALLY, N. J. & DE RONDE, J. (1981) Enhancement of the effect of cytotoxic drugs by radiosensitizers. *Br. J. Cancer*, **43**, 756.

MCNALLY, N. J. (1982) Enhancement of chemotherapy agents. *Int. J. Radiat. Oncol. Biol. Phys.*, **8**, 593.

MCNALLY, N. J., STEPHENS, T. C., TWENTYMAN, P. R. and 3 others (1982) The effect of cytoxic drugs with or without misonidazole on leucopenia in three strains of mice. *Int. J. Radiat. Oncol. Biol. Phys.*, **8**, 659.

ROJAS, A., STEWART, F. A. & DENEKAMP, J. (1982) Interaction of radiosensitizers and WR2721. 1. Modification of skin radioprotection. *Br. J. Cancer*, **45**, 684.

STEWART, F. A. & ROJAS, A. (1982) Radioprotection of mouse skin by WR2721 in single and fractionated treatments. *Br. J. Radiol.*, **55**, 42.

TRAVIS, E. L., DE LUCA, A. M., FOWLER, J. F. & PAKIOAL, T. N. (1982) The time course of radioprotection by WR2721 in mouse skin. *Int. J. Radiat. Oncol. Biol. Phys.*, **8**, 843.

TWENTYMAN, P. R. (1981) Modification of tumour and host response to cyclophosphamide by misonidazole and by WR2721. *Br. J. Cancer*, **43**, 745.

WASSERMAN, T. H., PHILLIPS, T. L., ROSS, G. & KANE, J. L. (1981) Differential protection against cytoxic chemotherapy effects in bone marrow CFU's by WR2721. *Cancer Clin. Trials*, **4**, 3.

YUHAS, J. M. (1979) Differential protection of normal and malignant tissues against the cytoxic effects of mechlorethamine. *Cancer Treat. Rep.*, **63**, 971.

YUHAS, J. M. (1980) Active *versus* passive absorption kinetics as the basis for selective protection of normal tissues by S-2-(3 aminopropylamino)-ethyl phosphorothioic acid. *Cancer Res.*, **40**, 1519.

YUHAS, J. M. & CULO, F. (1980) Selective inhibition of the nephrotoxicity of cis-dichlorodiammine-platinum by WR2721 without altering its anti-tumour properties. *Cancer Treat. Rep.*, **64**, p. 57.

YUHAS, J. M., SPELLMAN, J. M., JORDAN, S. W., PARDINI, M. C., AFZAD, S. M. J. & CULO, F. (1980) Treatment of tumours with the combination of WR2721 and cis-dichlorodiamine platinum (II) or cyclophosphamide. *Br. J. Cancer*, **42**, 574.